# Delineating Species with DNA Barcodes: A Case of Taxon Dependent Method Performance in Moths

**DOI:** 10.1371/journal.pone.0122481

**Published:** 2015-04-07

**Authors:** Mari Kekkonen, Marko Mutanen, Lauri Kaila, Marko Nieminen, Paul D. N. Hebert

**Affiliations:** 1 Finnish Museum of Natural History, University of Helsinki, Zoology Unit, University of Helsinki, Helsinki, Finland; 2 Biodiversity Institute of Ontario, University of Guelph, Guelph, Ontario, Canada; 3 Department of Genetics and Physiology, University of Oulu, Oulu, Finland; 4 Metapopulation Research Centre, Department of Biosciences, University of Helsinki, Helsinki, Finland; University of Veterinary Medicine Hanover, GERMANY

## Abstract

The accelerating loss of biodiversity has created a need for more effective ways to discover species. Novel algorithmic approaches for analyzing sequence data combined with rapidly expanding DNA barcode libraries provide a potential solution. While several analytical methods are available for the delineation of operational taxonomic units (OTUs), few studies have compared their performance. This study compares the performance of one morphology-based and four DNA-based (BIN, parsimony networks, ABGD, GMYC) methods on two groups of gelechioid moths. It examines 92 species of Finnish Gelechiinae and 103 species of Australian Elachistinae which were delineated by traditional taxonomy. The results reveal a striking difference in performance between the two taxa with all four DNA-based methods. OTU counts in the Elachistinae showed a wider range and a relatively low (ca. 65%) OTU match with reference species while OTU counts were more congruent and performance was higher (ca. 90%) in the Gelechiinae. Performance rose when only monophyletic species were compared, but the taxon-dependence remained. None of the DNA-based methods produced a correct match with non-monophyletic species, but singletons were handled well. A simulated test of morphospecies-grouping performed very poorly in revealing taxon diversity in these small, dull-colored moths. Despite the strong performance of analyses based on DNA barcodes, species delineated using single-locus mtDNA data are best viewed as OTUs that require validation by subsequent integrative taxonomic work.

## Introduction

After little progress over a long interval, the past decade has seen the development of several analytical methods which employ DNA sequences to delimit species boundaries [1–10]. Another innovation, DNA barcoding [11,12], was originally developed for specimen identification using a standardized segment of the mitochondrial genome (a 648bp region of the cytochrome *c* oxidase subunit I, COI), but its utility for species discovery was soon recognized [13–16]. The coupling of novel analytical methods with the rapid increase in data provided by DNA barcoding is creating a tremendous opportunity for taxonomists and biodiversity scientists. Large barcode datasets enable the delineation of hundreds or even thousands of putative species (i.e., operational taxonomic units, OTUs) simultaneously, allowing species recognition to proceed far more rapidly than through morphological approaches. Faced with accelerating losses of biodiversity, this increase in the efficiency of taxonomic workflows is acutely needed. Initial OTU delineation generates a good estimate of species diversity and provides a framework for subsequent taxonomic revisions (e.g., [17]).

Some methods available for species delineation are inappropriate for use with single-locus data (e.g., bpp [5]). Other methods, those requiring *a priori* defined groups (e.g., Population Aggregation Analysis [18]), cannot be employed for species discovery. However, a number of analytical approaches can be used for species delineation with single-locus data and they can be divided into three primary categories: clustering, tree-based and character-based. Clustering methods, the dominant category, employ diverse algorithms to recognize boundaries in distance matrices. This category includes, for instance, statistical parsimony networks (referred to here as TCS [19,20]), jMOTU [7], Clustering 16S rRNA for OTU Prediction (CROP [6]), Automatic Barcode Gap Discovery (ABGD [8]), and Barcode Index Number (BIN [9]). By comparison, tree-based methods, such as the Generalized Mixed Yule Coalescent (GMYC [3,4,21]), and Poisson Tree Processes (PTP [10]), employ a gene tree as input for the analysis. The third category, character-based methods, employs diagnostic base substitutions as a basis for decisions. To our knowledge, Character Attribute Organization System, CAOS [22–24] is the only available character-based method for testing species boundaries, although it also requires *a priori* defined groups so it cannot be used for their discovery. Cluster and tree-based approaches have become the dominant approaches used in studies of species delineation (bacteria [25], corals [26], molluscs [27–33], millipedes [34], spiders [35], insects [36–43], amphibians [44], bats [45], orchids [46]).

The relative performance of differing algorithmic approaches to species delineation has been examined in a few past studies. For example, it has been noted that GMYC produces more OTUs than TCS or ABGD [3,8,47,28,36,37] (but see [39]). When clusters recognized by GMYC have been compared with morphospecies, the conclusions have been variable. Early results showed high congruence between morphology and GMYC [3,4,36], but subsequent studies have indicated that GMYC often delivers a higher species count than morphology [29,44,45,48]. The largest comparison to date [9] examined one tree-based (GMYC) and four clustering (BIN, ABGD, jMOTU, CROP) methods with eight datasets comprising over 3000 species and close to 19000 DNA barcode sequences. This study revealed high performance for all methods with BIN slightly outperforming the other clustering methods, but similar to GMYC. In accordance with other studies, GMYC produced more splits than alternate methods. Zhang *et al*. [10] proposed that tree-based methods should outperform clustering methods in species assemblages lacking a ‘barcode gap’, the break between intra- and interspecific pairwise distances that underpins the success of DNA barcoding [12]. The lack of a gap is generally linked to recently diverged species with little genetic diversification, often coupled with incomplete lineage sorting and introgression [49,50]. In addition, it should be noted that incomplete lineage sorting and/or introgression generally lead to the failure of all methods based on the analysis of mitochondrial sequence divergences.

Although previous studies have provided a basic understanding of the performance of various species delineation methods, their behavior with different taxonomic groups has seen little investigation. The datasets examined by Ratnasingham and Hebert [9] involved large taxonomic assemblages (e.g., moths and butterflies of eastern North America) or comprehensive taxon coverage for a particular geographic region (e.g., geometrid moths of Bavaria). Their results showed some differences among higher taxa (e.g., North American plusiine moths), but much more detailed investigation of these effects is merited. This study extends past work by comparing the performance of five methods with two groups of moths, Finnish gelechiines and Australian elachistines. The Finnish gelechiine fauna includes 180 species belonging to 49 genera ([51], J. Kullberg, pers. comm.), while the Australian elachistines include 148 species in three genera [52]. Both subfamilies are members of the Gelechioidea, one of the largest radiations within the Lepidoptera, and provide a well-defined set of reference species established following detailed morphological and ecological studies [52–54]. Because these moths are generally small and dull-colored, they are very difficult for taxonomic studies so there are many undescribed species [55–57]. The two groups do have one difference; the Australian elachistines present a challenge for DNA barcoding due to their close affinities and supposed recent origins [58], while barcodes have successfully discriminated many European gelechiines (e.g., [14,59,60]). The adoption of DNA barcode-based methods for the delineation of species in the Gelechioidea and other taxa sharing their biological attributes has great potential to accelerate species delineation. Executing identical analyses with two datasets, one more challenging than the other, provides an opportunity to evaluate the performance of the differing methods in this context.

This study employs four commonly used methods for DNA-based species delineation, including one older but still widely used approach, statistical parsimony networks (TCS), and three recent methods: Barcode Index Numbers (BIN), Automatic Barcode Gap Discovery (ABGD) and Generalized Mixed Yule Coalescent (GMYC). These methods were selected for inclusion based on their general popularity and their strong performance in a previous study [9]. BIN analysis always generates only one number of OTUs for each set of DNA sequences, while the other approaches do not because key parameter values (TCS and ABGD) or input trees (GMYC) can vary. In addition to comparing the performance of these DNA-based approaches, we obtained results from a morphology-based analysis using external characters. We study differences between the outcomes for two datasets, considering both the count and composition of the putative species (OTUs) produced by each analysis. Finally, we evaluate the performance of the methods with singletons, as well as monophyletic and non-monophyletic (i.e., para- and polyphyletic) species.

## Materials and Methods

### Taxon sampling

Specimens of 92 species of Australian Elachistinae were sampled from the Australian National Insect Collection (ANIC) and the Finnish Museum of Natural History (MZH). Specimens of 103 species of Finnish Gelechiinae (tribes Teleiodini, Gelechiini and Gnorimoschemini) were sampled from the private collection of M.M. during 2008–2012 (17 of the latter specimens were collected from Denmark, Estonia, France, Latvia, Russia and Sweden). One additional elachistine specimen was analyzed from the Agricultural Scientific Collections Unit (ASCU), and three specimens of gelechiines from the private collection of Erkki Laasonen. Three to five specimens per species were usually sampled when available, targeting recently collected individuals from diverse geographic localities. Relatively few specimens of each taxon were analysed to maximize the species coverage. Larger sample sizes were examined for a few species whose taxonomic status is controversial. One or two legs were removed from dry pinned specimens for DNA extraction. Specimens were identified by L.K. (Elachistinae) and M.M. (Gelechiinae) following the taxonomy of Kaila [52] and Huemer and Karsholt [53,54], respectively. BOLD Sample and Process IDs, GenBank accession numbers and other details of our sequence data can be retrieved from S1 Table.

### DNA extraction, PCR amplification and sequencing

DNA extraction, PCR, and sequencing were performed at the Canadian Centre for DNA Barcoding following standard high-throughput protocols [61]. The first round of PCR employed the primers LepF1 and LepR1 [11] which generate a 658bp amplicon that spans the barcode region of COI. In cases of failure, two additional PCR reactions were carried out to recover 306bp and 407bp amplicons using a standard primer set [62]. If one of these reactions was successful, an effort was made to obtain a barcode compliant record (>497bp) by amplifying shorter regions of COI using the primer sets described in Hebert *et al*. [63]. All sequences were aligned using the BOLD Aligner in the Barcode of Life Data Systems (BOLD [64]) and then inspected visually for stop codons and frameshift mutations in MEGA5 [65].

### Comparison between datasets

Several attributes were studied to expose differences between the two datasets. Intra- and interspecific pairwise distances were calculated in the BOLD workbench employing the “Barcode Gap Analysis” tool, and visualized using the “sppDist” function in SPIDER [66] available in R [67]. The incidence of monophyly was quantified using the “monophyly” function of SPIDER. Pairwise distances for all sequences included in the analysis were calculated using a K2P distance model in MEGA5.

### Morphological sorting

In order to simulate the process of species recognition through morphological sorting, we recruited an experienced lepidopterist (M.N.) without previous knowledge of Australian elachistines or Finnish gelechiines to sort pinned specimens into OTUs using external morphology, mainly wing patterns, an approach similar to that employed in previous studies (e.g., [43]). The test collection included from one to five specimens of 96 species of Elachistinae and 83 species of Gelechiinae with representatives of most species that were used for DNA barcoding and a few additional taxa (Table 1). The individuals included were not DNA barcode vouchers, but other similarly identified museum specimens.

**Table 1:** Reference species, their monophyly on a DNA barcode gene tree, and the match of OTU composition in four DNA-based methods (BIN, TCS with 95% cut-off, GMYC with two Bayesian starting trees, ABGD with K2P, *X* = 1, Initial partition) and sorting relying on external morphology.

Dataset	Species	Monophyly	BIN	TCS 95%	GMYC Yule	GMYC Coal.	ABGD K2P	Morpho
Gelechiinae	*Altenia perspersella*	mono	M	M	M	M	M	MIX
	*Aroga velocella*	mono	M	M	M	M	M	MIX
	*Athrips amoenella*	mono	M	M	M	M	M	M
	*Athrips mouffetella*	mono	M	M	M	M	M	M
	*Athrips pruinosella*	mono	S	S	M	M	M	S
	*Athrips tetrapunctella*	mono	M	M	M	M	M	ME
	*Carpatolechia alburnella*	mono	M	M	M	M	M	S
	*Carpatolechia decorella*	singleton	M	M	M	M	M	N/A
	*Carpatolechia epomidella*	mono	M	M	M	M	M	M
	*Carpatolechia fugitivella*	mono	M	M	M	M	M	MIX
	*Carpatolechia notatella*	mono	M	M	M	M	M	MIX
	*Carpatolechia proximella*	mono	M	M	M	M	M	M
	Caryocolum amaurella*	mono	S	S	S	S	S	ME
	*Caryocolum blandella*	mono	M	M	M	M	M	N/A
	*Caryocolum blandelloides*	mono	M	M	M	M	M	ME
	*Caryocolum blandulella*	mono	M	M	M	M	M	MIX
	*Caryocolum cassella*	mono	M	M	M	M	M	ME
	*Caryocolum cauligenella*	mono	M	M	M	M	M	MIX
	*Caryocolum fischerella*	mono	M	M	M	M	M	MIX
	*Caryocolum fraternella*	mono	M	M	M	M	M	MIX
	*Caryocolum junctella*	singleton	M	M	M	M	M	MIX
	*Caryocolum kroesmanniella*	mono	M	M	M	M	M	MIX
	*Caryocolum petrophila*	mono	M	M	M	M	M	MIX
	*Caryocolum petryi*	mono	M	M	M	M	M	M
	*Caryocolum pullatella*	mono	M	M	M	M	M	MIX
	*Caryocolum schleichi*	mono	M	M	M	M	M	S
	*Caryocolum tischeriella*	singleton	M	M	M	M	M	M
	*Caryocolum tricolorella*	mono	M	M	M	M	M	S
	*Caryocolum vicinella*	mono	M	M	M	M	M	M
	*Caryocolum viscariella*	mono	M	M	M	M	M	MIX
	*Chionodes continuella*	mono	M	M	M	M	M	MIX
	Chionodes distinctella*	mono	S	S	S	S	M	MIX
	*Chionodes electella*	mono	M	M	M	M	M	MIX
	Chionodes fumatella*	non-mono	S	S	S	S	S	MIX
	*Chionodes holosericella*	mono	M	M	M	M	M	ME
	*Chionodes ignorantella*	mono	M	M	M	M	M	N/A
	*Chionodes luctuella*	mono	M	M	M	M	M	S
	*Chionodes lugubrella*	mono	M	M	M	M	M	S
	*Chionodes nubilella*	mono	M	M	M	M	M	ME
	*Chionodes tragicella*	mono	M	M	M	M	M	M
	*Chionodes viduella*	mono	M	S	S	M	M	M
	*Chionodes violacea*	mono	M	M	M	M	M	MIX
	*Cosmardia moritzella*	mono	M	M	M	M	M	N/A
	*Exoteleia dodecella*	mono	M	M	M	M	M	MIX
	*Filatima incomptella*	mono	M	M	M	M	M	M
	*Gelechia cuneatella*	mono	M	M	M	M	M	M
	*Gelechia hippophaella*	mono	M	M	M	M	M	M
	*Gelechia jakovlevi*	mono	M	M	M	M	M	M
	*Gelechia muscosella*	mono	M	M	M	M	M	ME
	*Gelechia nigra*	mono	M	M	M	M	M	MIX
	*Gelechia rhombella*	mono	M	M	M	M	M	S
	*Gelechia sabinellus*	mono	M	M	M	M	M	S
	*Gelechia sestertiella*	mono	M	M	M	M	M	M
	*Gelechia sororculella*	mono	M	M	M	M	M	M
	*Gelechia turpella*	mono	M	M	M	M	M	M
	*Gnorimoschema epithymella*	mono	M	M	M	M	M	MIX
	Gnorimoschema herbichii*	mono	S	S	S	S	M	M
	*Gnorimoschema nordlandicolella*	mono	M	M	M	M	M	MIX
	*Gnorimoschema streliciella*	mono	M	M	M	M	M	MIX
	*Gnorimoschema valesiella*	mono	M	M	M	M	M	ME
	*Klimeschiopsis kiningerella*	mono	M	M	M	M	M	ME
	Neofriseria peliella*	mono	S	S	S	S	M	M
	*Neofriseria singula*	mono	M	M	M	M	M	N/A
	*Neotelphusa sequax*	mono	M	M	M	M	M	MIX
	*Parachronistis albiceps*	mono	M	M	M	M	M	N/A
	*Prolita sexpunctella*	mono	M	M	M	M	M	MIX
	*Pseudotelphusa paripunctella*	mono	M	M	S	M	M	M
	*Pseudotelphusa scalella*	mono	M	M	M	M	M	M
	*Psoricoptera gibbosella*	mono	M	M	M	M	ME	N/A
	*Psoricoptera speciosella*	mono	M	M	M	M	ME	N/A
	*Recurvaria leucatella*	mono	M	M	M	M	M	M
	*Scrobipalpa acuminatella*	mono	M	M	M	M	M	MIX
	Scrobipalpa artemisiella*	mono	ME	ME	M	ME	ME	MIX
	*Scrobipalpa atriplicella*	mono	M	M	M	M	M	ME
	Scrobipalpa bryophiloides*	mono	S	S	S	S	M	N/A
	*Scrobipalpa murinella*	mono	M	M	M	M	M	ME
	*Scrobipalpa nitentella*	mono	M	M	M	M	M	MIX
	*Scrobipalpa obsoletella*	mono	M	M	M	M	M	MIX
	*Scrobipalpa pauperella*	mono	M	M	M	M	M	MIX
	*Scrobipalpa proclivella*	singleton	M	M	M	M	M	N/A
	*Scrobipalpa salicorniae*	mono	M	M	M	M	M	S
	*Scrobipalpa samadensis*	mono	M	M	M	M	M	MIX
	Scrobipalpa stangei*	mono	ME	ME	M	ME	ME	S
	*Scrobipalpopsis petasitis*	mono	M	M	M	M	M	S
	*Scrobipalpula diffluella*	singleton	M	M	M	M	ME	ME
	*Scrobipalpula psilella*	mono	M	M	M	M	ME	MIX
	*Stenolechia gemmella*	mono	M	M	M	M	M	N/A
	*Teleiodes flavimaculella*	mono	M	M	M	M	ME	MIX
	*Teleiodes luculella*	mono	M	M	M	M	ME	S
	*Teleiodes wagae*	singleton	M	M	M	M	M	ME
	*Teleiodes vulgella*	mono	M	M	M	M	M	M
	*Teleiopsis diffinis*	mono	M	M	M	M	M	M
Elachistinae	*Elachista aepsera*	mono	M	M	M	M	M	ME
	*Elachista alacera*	singleton	M	M	M	M	M	ME
	*Elachista aluta*	singleton	ME	ME	M	M	M	N/A
	Elachista anolba*	singleton	ME	ME	ME	ME	ME	ME
	*Elachista aposematica*	singleton	M	M	M	M	M	N/A
	*Elachista asperae*	mono	M	M	S	M	S	MIX
	Elachista averta*	non-mono	ME	ME	ME	ME	ME	N/A
	*Elachista bidens*	singleton	M	M	M	M	M	N/A
	*Elachista campsella*	singleton	M	M	M	M	M	ME
	Elachista carcharota*	mono	S	S	S	S	S	ME
	Elachista catagma*	non-mono	ME	ME	ME	ME	ME	ME
	*Elachista catarata*	mono	M	M	M	S	S	MIX
	*Elachista cerebrosella*	mono	M	M	S	M	M	N/A
	*Elachista chilotera*	singleton	M	M	M	M	M	M
	*Elachista coalita*	mono	M	M	M	M	M	MIX
	*Elachista corbicula*	singleton	M	M	M	M	M	ME
	*Elachista crenatella*	mono	M	M	M	S	M	ME
	*Elachista crocospila*	mono	M	M	M	M	M	MIX
	*Elachista crumilla*	mono	M	M	M	M	M	S
	*Elachista cyanea*	mono	M	M	M	M	M	MIX
	*Elachista cycotis*	mono	M	M	M	M	M	ME
	Elachista cylistica* ^*1*^	singleton	ME	ME	ME	ME	ME	ME
	*Elachista cynopa*	mono	M	M	M	M	M	MIX
	*Elachista delira*	mono	M	M	M	M	M	ME
	Elachista deusta* ^*3*^	non-mono	ME	ME	ME	ME	ME	ME
	*Elachista dieropa*	mono	M	M	M	M	M	MIX
	*Elachista diligens*	mono	M	M	M	M	M	MIX
	Elachista discina*	mono	S	M	MIX	S	S	MIX
	Elachista effusi* ^*3*^	non-mono	ME	ME	ME	ME	ME	M
	*Elachista elaphria*	mono	M	M	M	M	M	MIX
	*Elachista epartica*	singleton	M	M	M	M	M	N/A
	*Elachista eriodes*	mono	M	ME	M	M	M	M
	Elachista etorella* ^*1*^	non-mono	MIX	MIX	MIX	MIX	MIX	MIX
	*Elachista euthema*	singleton	M	M	M	M	M	N/A
	*Elachista evexa*	mono	M	M	M	S	M	ME
	Elachista faberella* ^*1*^	mono	ME	ME	ME	ME	ME	M
	*Elachista filiphila*	mono	M	M	M	M	M	M
	*Elachista flammula*	mono	M	M	M	M	M	S
	Elachista flavicilia* ^*3*^	non-mono	ME	ME	ME	ME	ME	MIX
	*Elachista floccella*	mono	M	M	M	M	M	MIX
	*Elachista fucosa*	singleton	M	M	M	M	M	ME
	*Elachista gemadella*	singleton	M	M	M	M	M	ME
	Elachista gerasmia*	non-mono	ME	ME	MIX	MIX	MIX	MIX
	Elachista gladiatrix* ^*1*^	mono	ME	ME	ME	ME	ME	MIX
	Elachista gladiograpta* ^*1*^	mono	ME	ME	ME	ME	ME	MIX
	*Elachista glomerella*	singleton	M	M	M	M	M	M
	*Elachista habrella*	singleton	M	M	M	M	M	MIX
	*Elachista ictera*	singleton	M	M	M	M	M	N/A
	*Elachista ignicolor*	mono	M	M	M	M	M	S
	*Elachista illota*	mono	M	ME	M	M	M	MIX
	Elachista lachnella* ^*2*^	mono	M	M	M	M	M	ME
	*Elachista levipes*	mono	M	M	M	M	M	ME
	*Elachista ligula*	singleton	M	M	M	M	M	MIX
	Elachista litharga*	non-mono	ME	ME	ME	ME	ME	MIX
	*Elachista magidina*	mono	M	M	M	M	M	MIX
	Elachista melanthes* ^*2*^	non-mono	MIX	MIX	MIX	MIX	MIX	M
	*Elachista menura*	mono	M	M	M	M	M	MIX
	*Elachista merista*	mono	M	M	M	M	M	ME
	*Elachista micalis*	singleton	M	M	M	M	M	ME
	*Elachista mundula*	mono	M	M	M	M	M	ME
	*Elachista mutarata*	mono	M	M	M	M	M	MIX
	*Elachista nielsencommelinae*	singleton	M	M	M	M	M	S
	Elachista nodosae* ^*2*^	non-mono	ME	ME	MIX	MIX	MIX	MIX
	*Elachista nr*. ophthalma*	singleton	ME	ME	ME	ME	ME	ME
	Elachista ophelma*	mono	ME	ME	ME	ME	ME	MIX
	Elachista ophthalma*	non-mono	ME	ME	ME	ME	ME	MIX
	*Elachista opima*	singleton	M	M	M	M	M	M
	Elachista paragauda* ^*1*^	non-mono	ME	ME	ME	MIX	ME	MIX
	Elachista paryphoea* ^*1*^	non-mono	ME	ME	ME	ME	ME	ME
	*Elachista patania*	singleton	M	M	M	M	M	N/A
	*Elachista patersoniae*	mono	M	M	M	S	M	S
	*Elachista peridiola*	mono	M	ME	M	M	M	M
	*Elachista pharetra*	singleton	M	M	M	M	M	M
	*Elachista phascola*	mono	M	ME	M	M	M	M
	Elachista physalodes*	non-mono	ME	ME	ME	ME	ME	MIX
	*Elachista platina*	mono	M	M	M	M	M	S
	Elachista platysma*	non-mono	ME	ME	MIX	ME	MIX	ME
	*Elachista polliae*	mono	M	M	M	M	M	ME
	*Elachista protensa*	singleton	M	M	M	M	M	N/A
	*Elachista ruscella*	singleton	M	M	M	M	M	MIX
	*Elachista sapphirella*	mono	M	M	M	M	M	MIX
	*Elachista sarota*	mono	M	M	M	M	M	ME
	*Elachista scitula*	singleton	M	M	M	M	M	N/A
	*Elachista seductilis*	mono	M	M	M	M	M	M
	*Elachista* sp. ANIC1	singleton	M	M	M	M	M	N/A
	*Elachista* sp. ANICLK1*	singleton	ME	ME	ME	M	M	N/A
	*Elachista* sp. ANICLK3*	singleton	ME	ME	ME	M	M	N/A
	*Elachista* sp. ANICLK4	singleton	M	M	M	M	M	N/A
	*Elachista* sp. ANICLK6	singleton	M	ME	M	M	M	N/A
	Elachista spathacea* ^*1*^	non-mono	ME	ME	ME	ME	ME	ME
	*Elachista sphaerella*	singleton	ME	ME	M	M	M	S
	*Elachista spinodora*	singleton	M	M	M	M	M	ME
	Elachista spongicola* ^*1*^	non-mono	ME	ME	MIX	ME	ME	MIX
	*Elachista stictifica*	mono	ME	ME	ME	ME	ME	MIX
	*Elachista strenua*	mono	M	M	M	M	M	MIX
	*Elachista synethes*	mono	M	M	M	S	S	MIX
	Elachista tetraquetri* ^*3*^	singleton	ME	ME	ME	ME	ME	N/A
	Elachista toralis* ^*1*^	mono	ME	ME	ME	ME	ME	M
	*Elachista toryna*	mono	M	M	M	M	M	MIX
	*Elachista velox*	mono	M	ME	M	M	M	MIX
	*Elachista velutina*	mono	M	M	M	M	M	MIX
	Elachista zophosema*	mono	ME	ME	ME	ME	ME	ME
	*Perittia daleris*	mono	M	M	M	M	M	ME

*Elachista aurita*, *E*. *cerina*, *E*. *chloropepla*, *E*. *commoncommelinae*, *E*. *festina*, *E*. *impiger*, *E*. *mystropa*, *E*. *propera*, *E*. *ravella*, *Neofaculta ericetella*, *Perittia antauges*, and *Pexicopia malvella* were used in sorting based on morphology, but were not included in the DNA-based delineation. M: MATCH, ME: MERGE, S: SPLIT, MIX: MIXTURE, mono: monophyletic, non-mono: either para- or polyphyletic.

### OTU delineation based on DNA barcodes

The Barcode Index Number System (BIN [9]), statistical parsimony networks (TCS [19,20]) and Automatic Barcode Gap Discovery (ABGD [8]) rely on pairwise sequence distances between specimens to determine the number of OTUs within a dataset. The RESL algorithm, which forms the basis of the BIN system, employs a three-stage procedure which starts with single linkage clustering using a fixed 2.2% threshold. This phase is followed by Markov clustering, which aims to improve the accuracy of the OTUs, and finally the Silhouette criterion compares the different clustering schemes from Markov clustering and chooses the option with the highest Silhouette score. ABGD employs a two-phase system which initially divides sequences into OTUs based on a statistically inferred barcode gap (i.e., initial partitioning), and subsequently conducts a second round of splitting (i.e., recursive partitioning). ABGD has three key parameters: (i) *X*, which is an estimate of relative gap width, and (ii) minimum and (iii) maximum values of prior intraspecific divergence (*P*), which are used to detect the barcode gap. The default *P*-values typically produce a range of OTU counts. TCS produces the most parsimonious solution for a particular cut-off value (90–99% cut-off values are available) by combining pairs of specimens with the lowest genetic distances. The procedure continues until the cut-off value is exceeded. The higher the cut-off, the smaller the number of steps needed to exceed it and the greater the count of unconnected networks recognized. In other words, selecting a high cut-off value produces a high species count and vice versa. The Generalized Mixed Yule Coalescent (GMYC [3,4,21]) differs strongly from the other methods because it is a model-based approach, aiming to discover the maximum likelihood solution for the threshold between the branching rates of speciation and coalescent processes on a tree. The number and composition of OTUs is inferred by counting the lineages crossing the threshold.

The BIN analysis was done using a stand-alone version of RESL which is scheduled for public release in the near future. Standard BIN assignments are available on BOLD v3.6 (http://www.boldsystems.org), but they are generated through the analysis of all barcode sequences on BOLD, meaning that the results are not strictly comparable with those obtained with other methods (because they are based on a more inclusive dataset). Statistical parsimony networks were calculated using software TCS v1.21 [20] with separate analyses for ten cut-off values (90%- 99%). ABGD analyses were performed on 24–25 March 2013 on the web interface (http://wwwabi.snv.jussieu.fr/public/abgd/). Because the default value for relative gap width (*X* = 1.5) did not produce a result for either dataset, two lower values (*X* = 0.8, 1.0) were used. 1.0 was the highest value that could be applied as ABGD did not produce results for the Gelechiinae dataset with *X* = 1.1. ABGD provides the option of using three distance metrics: Jukes-Cantor (JC [68]), Kimura 2 parameter (K2P [69]) and simple p-distances. We conducted analyses using all three metrics with both values (0.8, 1.0) of *X*, resulting in six analyses per dataset. All results using prior limits for intraspecific divergence ranging from *P* = 0.001–0.1 were recorded. Defaults were employed for all other parameter values.

GMYC requires a fully-resolved ultrametric chronogram as input. In order to test the effect of different input trees on GMYC, we calculated chronograms using three approaches: unweighted pair group method with arithmetic means (UPGMA [70]) and two Bayesian inference gene trees constructed with a Yule pure birth model [71,72] and constant size coalescent [73] tree priors. UPGMA trees have rarely been used with GMYC analyses [25,74], likely reflecting concerns with their effectiveness in phylogeny estimation (e.g., [75]), but they are an attractive option because of their speed and simplicity. The UPGMA trees used in this study were constructed in MEGA5 using a K2P distance model. Model selection for Bayesian analyses was performed *a priori* with jModeltest v.0.1.1 using the Akaike information criterion (AIC) [76] and *a posteriori* with Bayes factors implemented in Tracer v.1.5 [77]. GTR+G+I was the preferred model for Elachistinae with both methods, and for the Gelechiinae with Bayes factors, but not with jModeltest where HKY+G+I was preferred. As GTR+G+I was the second ranked option for jModeltest, the same model was employed for both datasets. The fit of clock models and tree priors were also estimated using Bayes factors with preference for the uncorrelated relaxed lognormal clock model over a strict clock and coalescent tree prior over Yule prior. Bayesian inference trees were constructed using BEAST [78,79]. XML files (S1–S4 Appendices) were made with the BEAUti v1.7.1 interface with the following settings: GTR+G+I substitution model; empirical base frequencies; 4 gamma categories; all codon positions partitioned with unlinked base frequencies and substitution rates. An uncorrelated relaxed lognormal clock model was used with rate estimated from the data and ucld.mean parameter with uniform prior employing 0 as the lower and 10 as the upper boundary. All other settings employed defaults. The length of the MCMC chain was 40 000 000 sampling every 4000. All BEAST runs were executed in BioPortal [80] and the resultant ESS values and trace files of runs were evaluated in Tracer. Two independent runs were combined using LogCombiner v.1.7.1 with 20% burn-in. Maximum clade credibility trees with 0.5 posterior probability limit and node heights of target tree were constructed in TreeAnnotator v1.7.1. Both single- and multiple-threshold GMYC analyses were conducted in R with the packages APE [81] and SPLITS [82]. All analyses related to GMYC were performed with haplotype data collapsed in ALTER [83].

### Direct examination of OTU composition

A simple comparison of OTU counts to the number of reference species can be misleading because similar results can be produced by varying levels of congruence between species and OTU boundaries if splits and merges are counterbalanced. In order to acquire a deeper insight, we estimated the correspondence between the boundaries of OTUs and reference species by assigning each OTU as a MATCH, SPLIT, MERGE or MIXTURE [9]. A MATCH results when the specimens assigned to an OTU include all those assigned to a reference species. By contrast, a SPLIT represents the case where members of a reference species are divided into two or more OTUs, while a MERGE represents the case where two or more reference species are assigned to a single OTU. MIXTURES involve more complex cases where members of two or more reference species are involved in both merger and splitting. Each OTU can only be assigned to one of these categories.

The performance of each method with singletons as well as with monophyletic and non-monophyletic species was studied by dividing datasets according to the results of the monophyly analysis by SPIDER (see Comparison between Datasets), and conducting a direct examination of congruence as described above. No additional OTU delineation analyses were executed with partitioned data.

## Results

A total of 562 full-length sequences (654bp; the original length 658bp is reduced by the BOLD aligner as it removes the first and three last bases) were recovered. These included 307 sequences (187 haplotypes) from 92 species in 25 genera of Gelechiinae and 255 sequences (178 haplotypes) from 103 species (including 6 undescribed species) in two genera of Elachistinae (Table 1). These datasets provide coverage for all Finnish gelechiine species in the tribes Teleiodini, Gelechiini and Gnorimoschemini, and for 65.5% of all Australian elachistines. We only included full-length sequences to minimize the possible effects of missing bases on the outcomes of subsequent analyses. All sequences are available in public databases (for GenBank accessions see S1 Table; BOLD dataset DS-GELEELA, DOI: 10.5883/DS-GELEELA). The number of samples per species varied from 1–19 in the Gelechiinae (mean = 3.34) and from 1–14 in the Elachistinae (mean = 2.47).

### Dataset comparison

**Fig 1:**
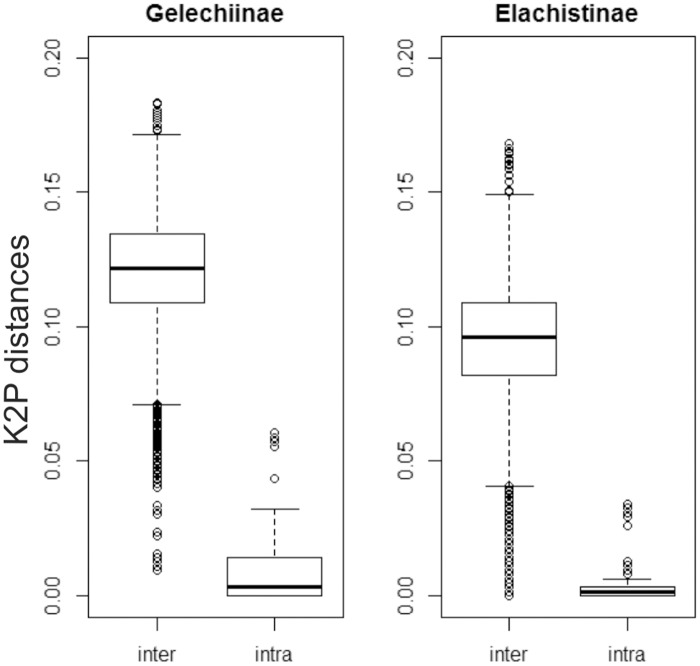
Intra- and interspecific distances (K2P) at COI for 92 species of Finnish Gelechiinae and 103 species of Australian Elachistinae.

**Fig 2:**
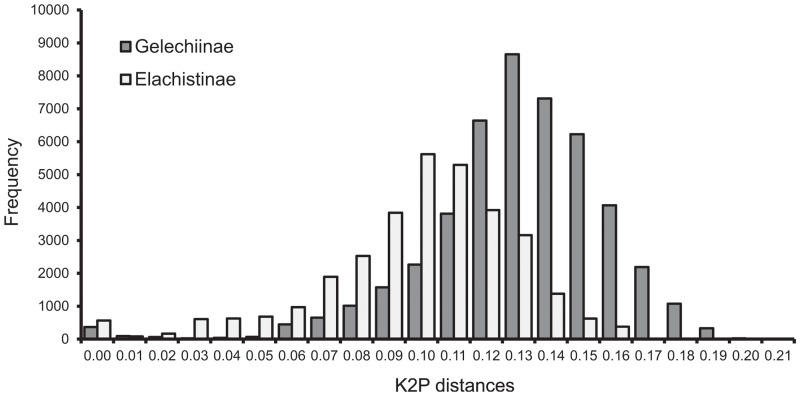
Pairwise distances (K2P) at COI without *a priori* grouping for 92 species of Finnish Gelechiinae and 103 species of Australian Elachistinae.

Intraspecific distances in the Gelechiinae varied from 0.00% to 2.94% (mean = 0.39%, SE = 0.01) while distance to the nearest neighbor (NN) species ranged from 0.92% to 11.25% (mean = 6.33%, SE = 0.02) so there were few cases of overlap between intra- and interspecific distances (Fig 1). A similar pattern was observed in the Elachistinae with intra-specific distances ranging from 0.00% to 2.3% (mean = 0.28%, SE = 0.01), while NN distances varied from 0.00% to 11.02% (mean = 3.48%, SE = 0.03) (Fig 1). Because these distance measures reflect past decisions on species boundaries (which may be incorrect), they may be biased, but this effect can be reduced by calculating pairwise distances without *a priori* grouping. This analysis confirmed that the average distance among all sequences was lower for Australian elachistines (mean = 0.099) than for Finnish gelechiines (mean = 0.13) (Fig 2). The proportion of monophyletic groups was also very different: 99% of the Gelechiinae species were monophyletic (85 monophyletic, 1 non-monophyletic, 6 singletons), but only 75% of the Elachistinae (52 monophyletic, 17 non-monophyletic, 34 singletons).

### Morphological sorting

**Fig 3:**
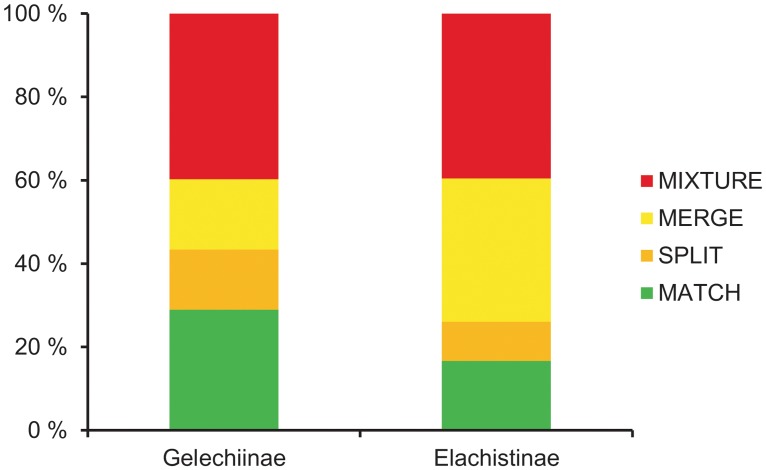
Sorting based on external morphology for 83 species of Finnish Gelechiinae and 96 species of Australian Elachistinae. OTU composition is evaluated against reference species.

The number of putative species resulting from morphological sorting was close to the reference count for both the Gelechiinae (91 vs. 83 reference species) and the Elachistinae (97 vs. 96). However, the composition of the OTUs showed a poor match with accepted taxonomy (Fig 3). Only 29% of the Gelechiinae species were correctly assigned, and 17% of the Elachistinae. As well, a very high proportion (40%) of the OTUs in both subfamilies represented MIXTURES of two or more species.

### OTU counts

**Fig 4:**
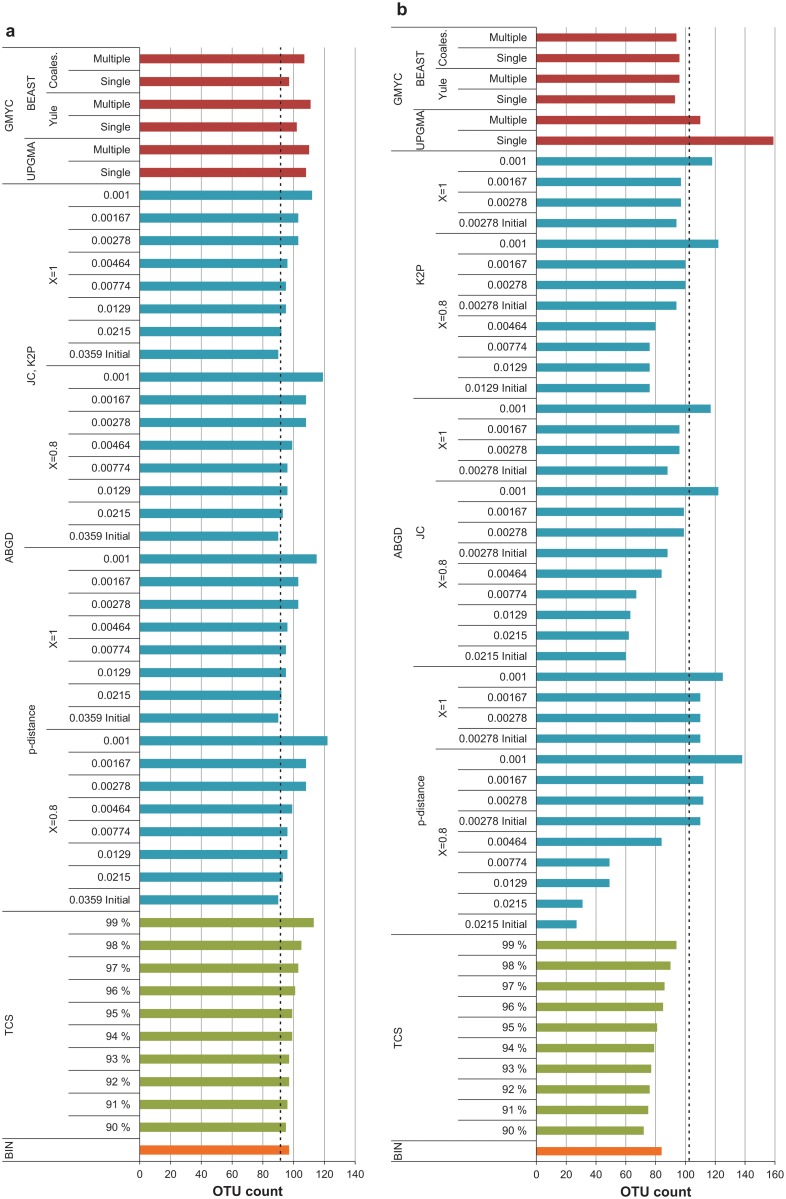
OTU counts for 92 species of Finnish Gelechiinae and 103 species of Australian Elachistinae sorted by methods. BIN, parsimony networks (TCS) with 90–99% cut-off values, ABGD with JC and K2P distance metrics, two *X*-values (0.8, 1) and a range of *P*-values (below the results), and GMYC with three starting trees (UPGMA, Bayesian with Yule and coalescent tree priors) and two models (single- and multiple-threshold). Dashed lines indicate reference species count (92/103). (a) Gelechiinae, (b) Elachistinae.

**Fig 5:**
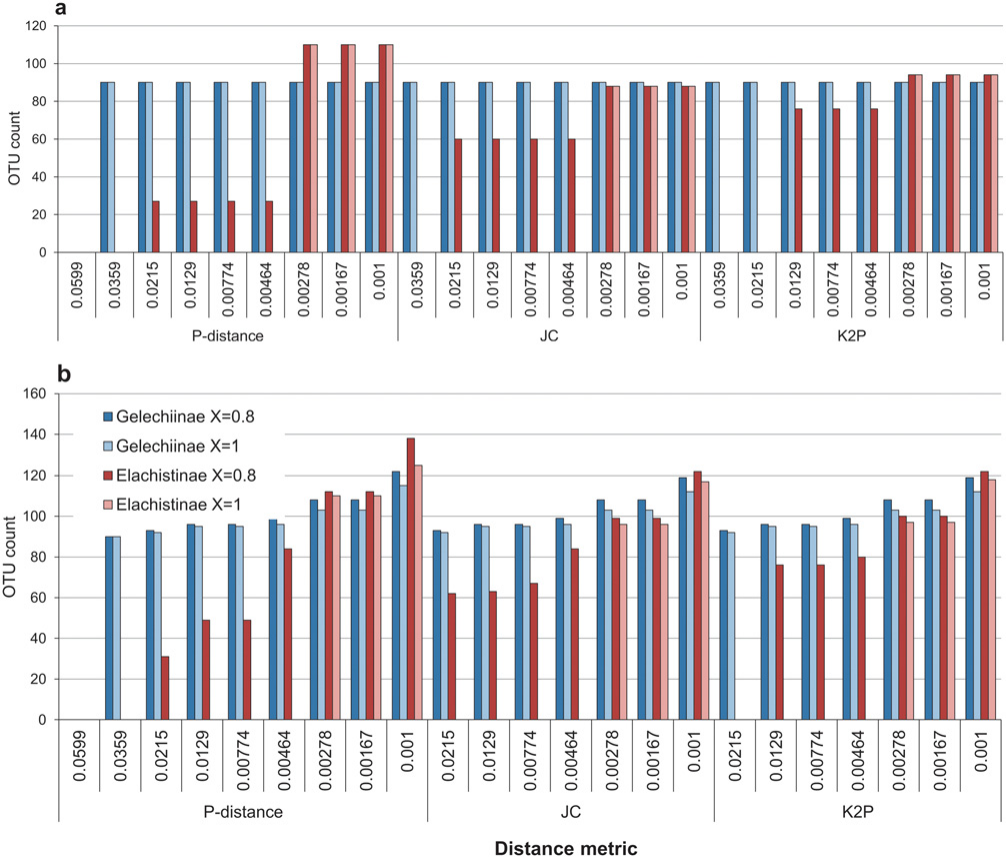
OTU counts for 92 species of Finnish Gelechiinae and 103 species of Australian Elachistinae resulting from ABGD. (a) Initial partitions, (b) recursive partitions. figures below the results indicate prior intraspecific divergence (*P*) values (in reverse order by distance metric).

**Fig 6:**
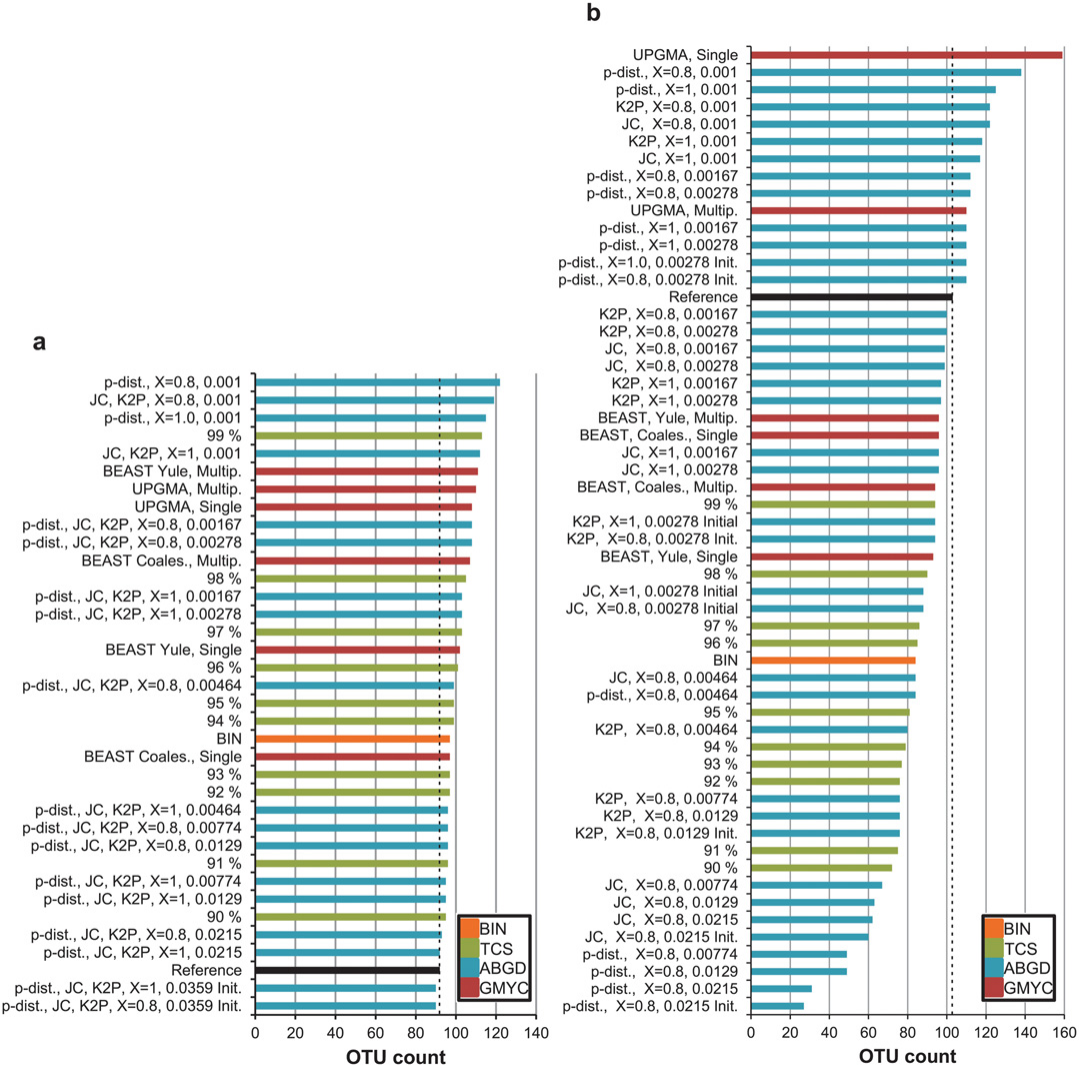
Ranked OTU counts for 92 species of Finnish Gelechiinae and 103 species of Australian Elachistinae. Black bars and dash lines show the reference species count. (a) Gelechiinae, (b) Elachistinae.

OTU counts produced by the DNA-based delimitation methods ranged from 90 to 122 for the Gelechiinae (Figs 4a, 5a and 5b) and from 27 to 159 for the Elachistinae (Figs 4b, 5a and 5b). Only one method generated the same OTU count as the number of reference species (92) for the Gelechiinae: Automatic Barcode Gap Discovery with relative gap width (*X* = 1.0) and prior intraspecific divergence (*P* = 0.0215). None of the methods generated the same OTU count as the number of reference species (103) for the Elachistinae. The relative number of OTUs versus the reference species count varied between the two datasets: most OTU counts for the Gelechiinae were higher than the reference count of 92 species (Fig 6a), whereas most for the Elachistinae were lower than the reference count of 103 species (Fig 6b).

BIN: BIN generated a single outcome for each dataset, recognizing 97 OTUs for the Gelechiinae, and 84 for the Elachistinae. (Fig 4a and 4b). In general, the BIN results followed the same pattern as the other methods with a higher OTU count than the reference sequence count for the Gelechiinae and a lower OTU count for the Elachistinae (Fig 4a and 4b). When compared with the other methods, the BIN results were approximately in the middle of the performance plots (orange bars in Fig 6a and 6b).

TCS: TCS was used with all available cut-off values (90–99%, resulting in ten outcomes. As expected, the lowest cutoff (90%) always generated the fewest clusters while the highest (99%) generated the most (Gelechiinae: 95–113, Elachistinae: 72–94) (Fig 4a and 4b). The relative position of the OTU counts differed between the two datasets: the TCS results were scattered among those for the other methods in the Gelechiinae, while those for the Elachistinae had low values (green bars in Fig 6a and 6b). All results from TCS were higher than the reference species count for the Gelechiinae, but lower for the Elachistinae (black vs. green bars in Fig 6a and 6b).

ABGD: ABGD was used with two values of relative gap width (*X*) and three distance metrics (p, JC, K2P). All OTU counts resulting from varying values of prior intraspecific divergence (*P*) were recorded (Fig 5a and 5b). All analyses produced zero OTUs for the Gelechiinae when *P* = 0.0599 and the initial partition with 90 OTUs was reached when *P* = 0.0359. All distance metrics behaved similarly with the Gelechiinae generating OTU counts ranging from 90 to 122 (*X* = 0.8) and from 90 to 115 (*X* = 1.0) (Figs 4a, 5a and 5b). The pattern changed with the Elachistinae dataset (Figs 4b, 5a and 5b). The two values of *X* produced differences for the initial partitions: either one (*X* = 1.0) or two (*X* = 0.8) OTU counts were generated by the initial partition (Fig 5a). ABGD behaved similarly with recursive partitions (Fig 5b), but there were differences between the three distance metrics. P-distance produced OTU counts ranging from 27 to 138 (*X* = 0.8) and from 110 to 125 (*X* = 1.0), while JC (*X* = 0.8: 60–122; *X* = 1.0: 88–117) and K2P (*X* = 0.8: 76–122; *X* = 1.0: 94–118) generated more constrained counts when *X* = 0.8 (Fig 5a). ABGD was the only method to produce the same OTU count as the number of reference species for the Gelechiinae with high prior intraspecific divergence value (*P* = 0.0215). By contrast, the closest match (100 OTUs) for the Elachistinae was generated by two low *P*-values (0.00278 and 0.00167) (Fig 6a and 6b).

GMYC: GMYC was used with three input trees: UPGMA and two Bayesian chronograms constructed in BEAST with Yule and coalescent tree priors. The results of both single- and multiple-threshold models were recorded (Table 2), although only one analysis indicated a better fit for the multiple-threshold model (UPGMA starting tree with Elachistinae; *χ* ^*2*^ = 93.22, d.f. = 21, *P*<<0.001). The likelihood ratio test was highly significant for all analyses, indicating rejection of the null model (OTU count = 1). The single-threshold model generally produced lower cluster counts than the multiple-threshold for the Gelechiinae and the starting trees constructed in BEAST resulted in lower counts than the UPGMA trees (Fig 4a). All GMYC analyses recognized more OTUs than the reference species count for the Gelechiinae (97–111 OTUs vs. 92 species) (purple bars in Fig 6a). GMYC behaved differently with the Elachistinae dataset. The single-threshold analysis based on an UPGMA starting tree recognized 159 OTUs, which was far more than the other analyses (Fig 4b). The multiple-threshold model based on the UPGMA tree also generated a high OTU count (110), whereas the GMYC analyses with Bayesian trees were more stable, recognizing from 93 to 96 OTUs (Fig 4b). The reference species count was between UPGMA and BEAST results (black vs. purple bars in Fig 6a and 6b).

**Table 2:** Results of the Generalized Mixed Yule Coalescent (GMYC) analyses.

Dataset	Input tree	Analysis	Clusters (CI)	Entities (CI)
Gelechiinae	UPGMA	Single	58 (56–58)	108 (103–111)
		Multiple	62 (62–62)	110 (96–110)
	BEAST, Yule	Single	61 (59–61)	102 (96–108)
		Multiple	65 (65–65)	111 (107–113)
	BEAST, Coalescent	Single	59 (57–60)	97 (93–109)
		Multiple	64 (56–64)	107 (89–109)
Elachistinae	UPGMA	Single	14 (14–14)	159 (159–159)
		Multiple	45 (42–45)	110 (108–111)
	BEAST, Yule	Single	43 (41–44)	93 (89–96)
		Multiple	44 (42–44)	96 (92–96)
	BEAST, Coalescent	Single	42 (41–45)	96 (81–98)
		Multiple	39 (38–49)	94 (77–94)

Clusters: OTUs delineated by GMYC with more than one specimen, Entities: singleton OTUs delineated by GMYC, CI: confidence interval, BEAST: Bayesian gene tree reconstructed in BEAST, Yule: Yule tree prior, Coalescent: coalescent tree prior, Single: single threshold model, Multiple: multiple threshold model.

### Method performance based on OTU composition

**Fig 7:**
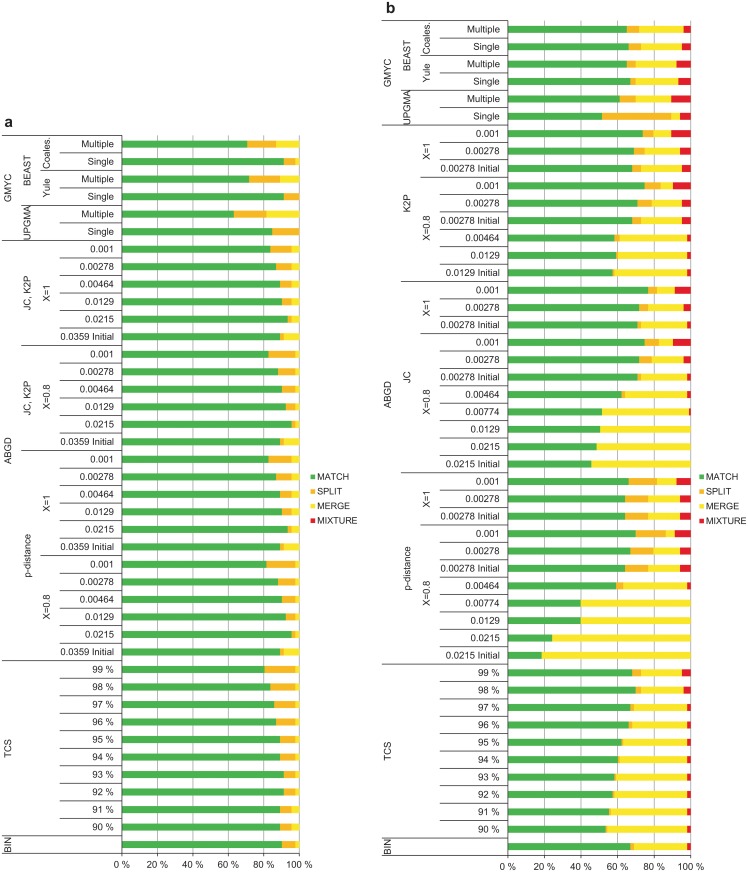
Method performance for 92 species of Finnish Gelechiinae and 103 species of Australian Elachistinae. (a) Gelechiinae, (b) Elachistinae.

The percentage of MATCHES was generally higher for the Gelechiinae (63–96%) than for the Elachistinae (46–77%; logistic regression: estimate = -1.29, n = 67, *P*<0.0001), reflecting the increased proportion of MERGES and MIXTURES for the Elachistinae (Fig 7a and 7b). The results generated by ABGD with p-distance were excluded from these statistical tests due to the strongly discordant results generated by this method for the Elachistinae (MATCH: 18–70%). The highest percentage of MATCHES was produced by ABGD for both datasets, but with very different *P*-values (Gelechiinae: p-distance, JC, K2P, *X* = 0.8, *P* = 0.0215; Elachistinae: JC, *X* = 1, *P* = 0.001) (Table 3, Fig 7a and 7b). ABGD also generated the lowest percentage of MATCHES for the Elachistinae (Fig 7b), while the poorest result for the Gelechiinae was produced by the multiple-threshold GMYC with UPGMA input tree (Fig 7a).

**Table 3:** Comparison of the performance of four analytical methods (ABGD, BIN, GMYC, TCS) ranked by the number of MATCHES.

Dataset	Method	Parameters		MATCH	SPLIT	MERGE	MIXTURE
Gelechiinae	GMYC	UPGMA	multiple	58	17	17	0
	GMYC	BEAST, coalescent	multiple	65	15	12	0
	GMYC	BEAST, Yule	multiple	66	16	10	0
	TCS		99%	74	16	2	0
	ABGD	p-distance, X = 0.8	P = 0.001	75	15	2	0
	ABGD	p-distance, X = 0.1	P = 0.001	76	12	4	0
	ABGD	JC, K2P, X = 0.8	P = 0.001	76	14	2	0
	TCS		98%	77	13	2	0
	ABGD	JC, K2P, X = 1.0	P = 0.001	77	11	4	0
	GMYC	UPGMA	single	78	14	0	0
	TCS		97%	79	11	2	0
	TCS		96%	80	10	2	0
	ABGD	p-distance, X = 1.0	P = 0.00278	80	8	4	0
	ABGD	JC, K2P, X = 1.0	P = 0.00278	80	8	4	0
	ABGD	p-distance, X = 0.8	P = 0.00278	81	9	2	0
	ABGD	JC, K2P, X = 0.8	P = 0.00278	81	9	2	0
	TCS		91%	82	6	4	0
	TCS		94%	82	8	2	0
	TCS		95%	82	8	2	0
	ABGD	p-distance, X = 0.8	P = 0.0359*	82	2	8	0
	ABGD	p-distance, X = 0.1	P = 0.0359*	82	2	8	0
	ABGD	JC, K2P, X = 0.8	P = 0.0359*	82	2	8	0
	ABGD	JC, K2P, X = 1.0	P = 0.0359*	82	2	8	0
	ABGD	p-distance, X = 1.0	P = 0.00464	82	6	4	0
	ABGD	JC, K2P, X = 1.0	P = 0.00464	82	6	4	0
	TCS		90%	83	5	4	0
	BIN			83	7	2	0
	ABGD	p-distance, X = 0.8	P = 0.00464	83	7	2	0
	ABGD	JC, K2P, X = 0.8	P = 0.00464	83	7	2	0
	ABGD	p-distance, X = 1.0	P = 0.0129	83	5	4	0
	ABGD	JC, K2P, X = 1.0	P = 0.0129	83	5	4	0
	TCS		92%	84	6	2	0
	TCS		93%	84	6	2	0
	GMYC	BEAST, Yule	single	84	8	0	0
	GMYC	BEAST, coalescent	single	84	6	2	0
	ABGD	p-distance, X = 0.8	P = 0.0129	85	5	2	0
	ABGD	JC, K2P, X = 0.8	P = 0.0129	85	5	2	0
	ABGD	p-distance, X = 1.0	P = 0.0215	86	2	4	0
	ABGD	JC, K2P, X = 1.0	P = 0.0215	86	2	4	0
	ABGD	p-distance, X = 0.8	P = 0.0215	88	2	2	0
	ABGD	JC, K2P, X = 0.8	P = 0.0215	88	2	2	0
Elachistinae	ABGD	p-distance, X = 0.8	P = 0.0215*	19	0	84	0
	ABGD	p-distance, X = 0.8	P = 0.0215	25	0	78	0
	ABGD	p-distance, X = 0.8	P = 0.0129	41	0	62	0
	ABGD	JC, X = 0.8	P = 0.0215*	47	0	56	0
	ABGD	JC, X = 0.8	P = 0.0215	50	0	53	0
	ABGD	JC, X = 0.8	P = 0.0129	52	0	51	0
	ABGD	JC, X = 0.8	P = 0.00774	53	0	49	1
	GMYC	UPGMA	single	53	39	5	6
	TCS		90%	55	1	45	2
	TCS		91%	57	1	43	2
	TCS		92%	59	1	41	2
	ABGD	K2P, X = 0.8	P = 0.0129*	59	1	41	2
	TCS		93%	60	1	40	2
	ABGD	K2P, X = 0.8	P = 0.00464	60	3	38	2
	ABGD	p-distance, X = 0.8	P = 0.00464	61	4	36	2
	ABGD	K2P, X = 0.8	P = 0.0129	61	1	39	2
	ABGD	K2P, X = 0.8	P = 0.00774	61	1	39	2
	TCS		94%	62	1	38	2
	GMYC	UPGMA	multiple	63	9	20	11
	TCS		95%	64	1	36	2
	ABGD	JC, X = 0.8	P = 0.00464	64	2	35	2
	ABGD	p-distance, X = 0.8	P = 0.00278*	66	13	18	6
	ABGD	p-distance, X = 1.0	P = 0.00278*	66	13	18	6
	ABGD	p-distance, X = 1.0	P = 0.00278	66	13	18	6
	GMYC	BEAST, coalescent	multiple	67	7	25	4
	GMYC	BEAST, Yule	multiple	67	5	23	8
	TCS		96%	68	2	31	2
	ABGD	p-distance, X = 1.0	P = 0.001	68	16	11	8
	GMYC	BEAST, coalescent	single	68	7	23	5
	ABGD	p-distance, X = 0.8	P = 0.00278	69	13	15	6
	TCS		97%	69	2	30	2
	BIN			69	2	30	2
	GMYC	BEAST, Yule	single	69	3	24	7
	TCS		99%	70	5	23	5
	ABGD	K2P, X = 0.8	P = 0.00278*	70	5	23	5
	ABGD	K2P, X = 1	P = 0.00278*	70	5	23	5
	ABGD	K2P, X = 1	P = 0.00278	71	6	20	6
	TCS		98%	72	3	24	4
	ABGD	p-distance, X = 0.8	P = 0.001	72	17	5	9
	ABGD	JC, X = 0.8	P = 0.00278*	73	2	26	2
	ABGD	JC, X = 1	P = 0.00278*	73	2	26	2
	ABGD	K2P, X = 0.8	P = 0.00278	73	8	17	5
	ABGD	JC, X = 0.8	P = 0.00278	74	7	18	4
	ABGD	JC, X = 1	P = 0.00278	74	5	20	4
	ABGD	JC, X = 1	P = 0.00167	74	5	20	4
	ABGD	K2P, X = 1	P = 0.001	76	6	10	11
	ABGD	JC, X = 0.8	P = 0.001	77	8	8	10
	ABGD	K2P, X = 0.8	P = 0.001	77	9	7	10
	ABGD	JC, X = 1	P = 0.001	79	5	10	9

BIN has a single OTU estimate for each dataset, while GMYC has 6 and TCS has 10. There are 36 outcomes for ABGD for the Gelechiinae (JC and K2P are combined as the results were identical) and 32 for the Elachistinae. Description of parameters and MATCH, SPLIT, MERGE and MIXTURE categories are provided in the Material and Methods.

BEAST: Bayesian gene tree reconstructed in BEAST, Yule: Yule tree prior, Coalescent: coalescent tree prior, Single: single threshold model, Multiple: multiple threshold model, JC: Jukes-Cantor substitution model, K2P: Kimura two parameter substitution model, X: relative gap width, P: prior intraspecific divergence value,

*: initial partition.

BIN: BIN produced a high percentage of MATCHES for the Gelechiinae (90%), but substantially less for the Elachistinae (67%).

TCS: TCS generated a varying proportion of MATCHES depending on the cut-off value. The highest percentage (91%) of MATCHES for the Gelechiinae was obtained with 92% and 93% cut-off values, while the best result (70%) for the Elachistinae was obtained with a cut-off value of 98%.

ABGD: The initial partition produced 89% MATCHES for the Gelechiinae irrespective of distance metric or value of relative gap width. The highest percentage of MATCHES was generated by *P* = 0.0215 (*X* = 0.8, 96%; *X* = 1.0, 93%) and the lowest by *P* = 0.001 (*X* = 0.8, 83%; *X* = 1.0, 84%). By contrast, the two values of relative gap width and the different distance metrics had clear effects on the performance of ABGD for the Elachistinae. The two initial partitions produced by *X* = 0.8 differed in their percentage of MATCHES (*P* = 0.001–0.00278: 64–71%; *P* = 0.00464–0.00215: 18–57%). *X* = 1.0 generated only one initial partition, which was the same as the partition with lower *P*-values of *X* = 0.8. JC (46–77%) and K2P (57–75%) generated a similar percentage of MATCHES, although JC was more variable. P-distance performed very poorly (18–70%), especially with *P*-values from 0.00464 to 0.0215. The most congruent outcome with the reference species (77% MATCHES) was produced by *P* = 0.001 with JC and *X* = 1, but the same *P*-value generated also the second highest percentage of MATCHES with K2P and both values of *X*.

GMYC: The single-threshold model in GMYC clearly outperformed multiple-threshold model when used with the Bayesian input trees. The pattern was very similar for both datasets, but the difference was larger for the Gelechiinae (single-threshold: 91% MATCHES, multiple-threshold: 71–72%) than for the Elachistinae (single-threshold: 66–67%, multiple-threshold: 65%). The performance of the UPGMA starting tree was weaker than the Bayesian gene trees for both datasets, but the tree priors caused only a minor difference (Gelechiinae: UPGMA, single-threshold: 85%, multiple-threshold: 63%; BEAST with Yule tree prior, single-threshold: 91%, multiple-threshold: 72%; BEAST with coalescent tree prior, single-threshold: 91%, multiple-threshold: 71%; Elachistinae: UPGMA, single-threshold: 51%, multiple-threshold: 61%; BEAST with Yule tree prior, single-threshold: 67%, multiple-threshold: 65%; BEAST with coalescent tree prior, single-threshold: 66%, multiple-threshold: 65%).

### Singletons, mono- and non-monophyletic species

**Fig 8:**
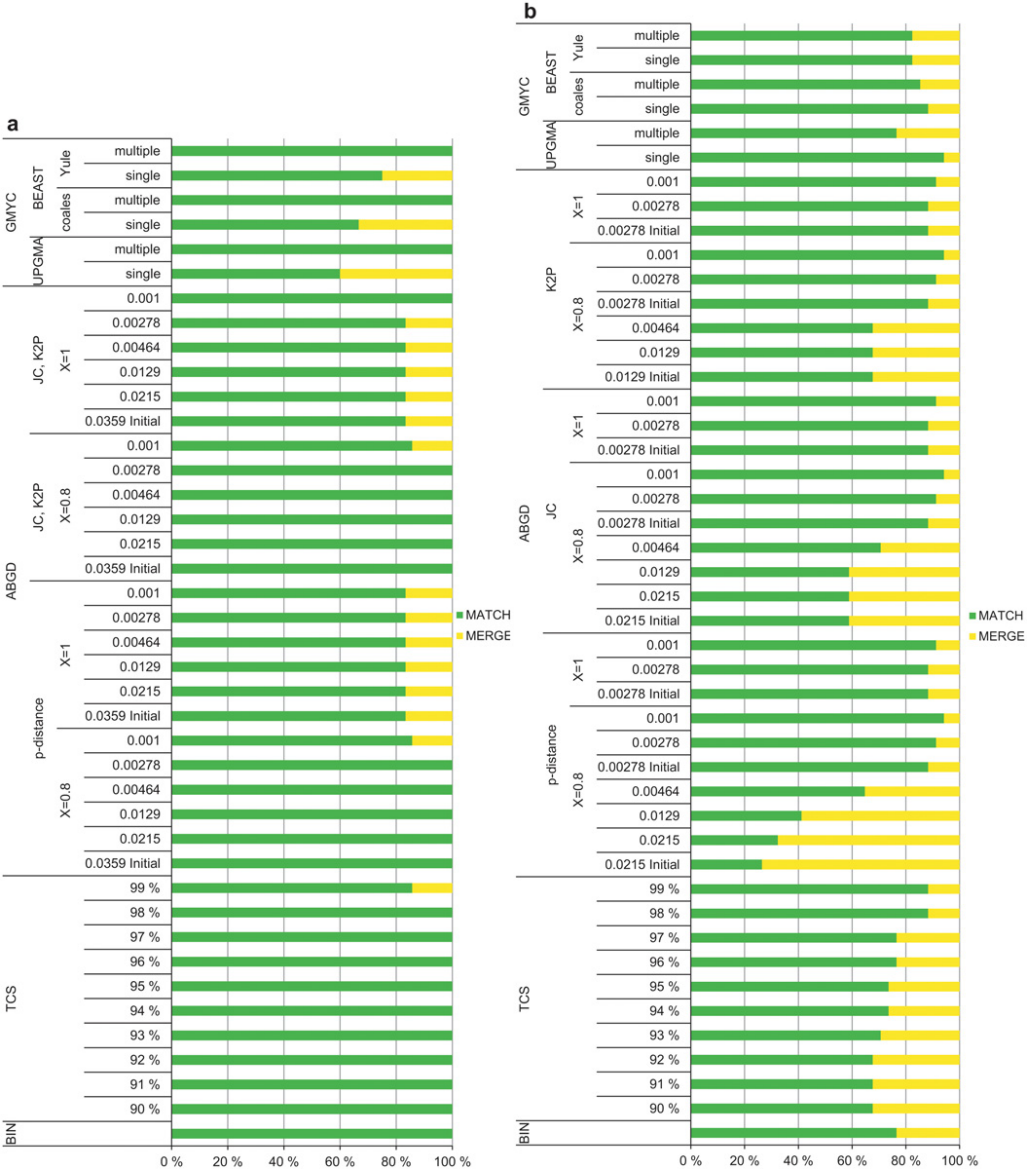
Performance with singletons for 6 species of Finnish Gelechiinae and 34 species of Australian Elachistinae. (a) Gelechiinae, (b) Elachistinae.

Most singletons in the Gelechiinae dataset matched with their corresponding reference species (BIN, all TCS, all GMYC with single-threshold, most ABGD with *X* = 0.8: 100%), whereas the percentage of MATCHES varied much more for the Elachistinae (Fig 8a and 8b). BIN and TCS with 95% cut-off produced a similar percentage of MATCHES for the Elachistinae (74% and 76%, respectively). Other results of TCS varied from 68% (90% cut-off) to 88% (99% cut-off). The highest percentage of MATCHES for the Elachistinae was produced by GMYC with UPGMA starting tree and single-threshold model (94%) (S2 Table); the rest varied from 76% (UPGMA, multiple-threshold) to 88% (BEAST with coalescent prior, single-threshold) (Fig 8a and 8b). The results of ABGD spanned a wide range from 26% (p-distance, *X* = 0.8, *P* = 0.0215 Initial) to 94% (JC, *X* = 0.8, *P* = 0.001; K2P, *X* = 0.8, *P* = 0.001) (Fig 8a and 8b).

**Fig 9:**
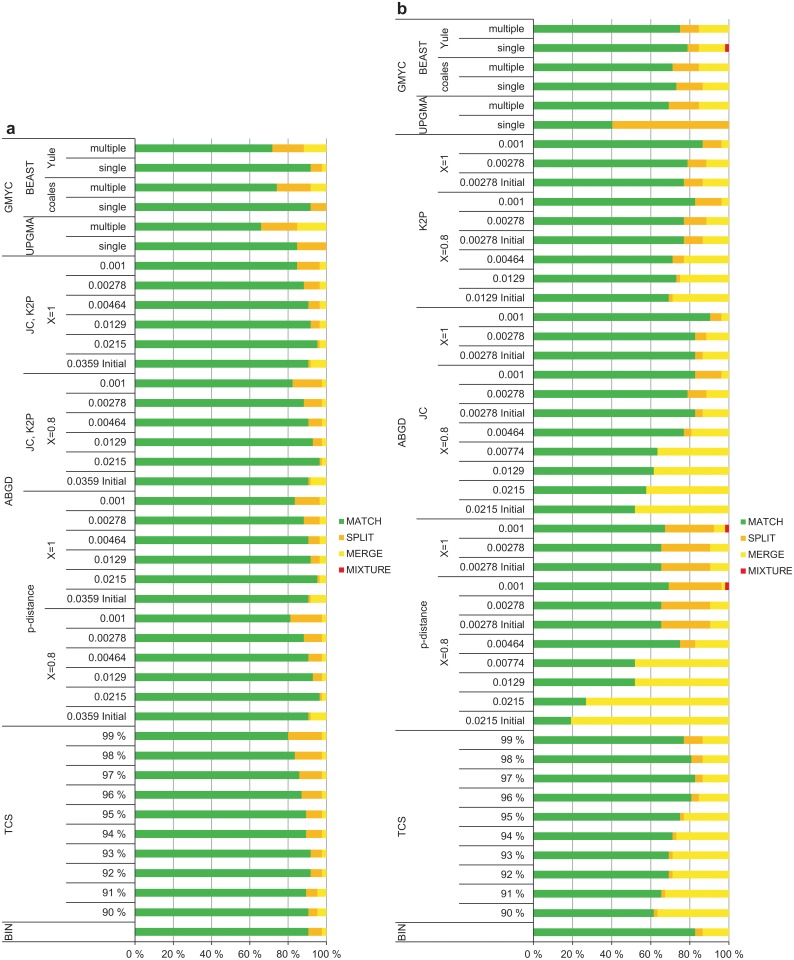
Performance with monophyletic species for 85 species of Finnish Gelechiinae and 52 species of Australian Elachistinae. (a) Gelechiinae, (b) Elachistinae.

The examination of mono- and non-monophyletic (i.e., para- or polyphyletic) species revealed that none of the methods was effective in delimiting non-monophyletic taxa. The only exception was ABGD with small *P*-values, which managed to deliver one or two MATCHES. On the other hand, ABGD also produced a high number of MIXTURES together with these MATCHES. As only one species in the Gelechiinae dataset was non-monophyletic and six were singletons, the difference was minor between the analyses including all data and only monophyletic species (all data: mean = 86.5%; monophyletic: mean = 87.3%; logistic regression: estimate = 0.07, n = 58, *P* = 0.4207). However, the performance was significantly improved for the Elachistinae (all data: mean = 63.9%; monophyletic: mean = 73.9%; logistic regression: estimate = 0.47, n = 76, *P*<0.0001) (Fig 9a and 9b). The taxon-dependent pattern in general performance remained even after removing all non-monophyletic species and singletons as the percentage of MATCHES was still significantly higher for the Gelechiinae than for the Elachistinae dataset (Fig 9a and 9b; logistic regression: estimate = -0.89, n = 67, *P*<0.0001). The performance of individual methods when non-monophyletic species and singletons were excluded was rather similar to the performance revealed by all data (Fig 9a, 9b and S3 Table).

## Discussion

This study has compared the performance of five species delineation methods (BIN, TCS, ABGD, GMYC, and external morphology) with two groups of Lepidoptera: Finnish Gelechiinae and Australian Elachistinae. The difference between the groups was evident as the Gelechiinae had a wider barcode gap (Fig 2) and included more monophyletic species than the Elachistinae. The Gelechiinae also seem more morphologically diverse as indicated by their assignment to 25 genera, while all but one of the 92 species of Australian elachistines are in a single genus. Our results reveal a striking difference between the two taxa in the effectiveness of varied delineation methods in recovery of current species boundaries. Performance was generally higher for all methods with the Gelechiinae than the Elachistinae. The range between the lowest and highest OTU counts was smaller, and the percentage of MATCHES was also higher for the Gelechiinae than for the Elachistinae. The higher proportion of MATCHES for the Gelechiinae remained evident when monophyletic species and singletons were studied separately, although the performance improved in all methods.

### Explaining the differential success in species delineation between Gelechiinae and Elachistinae

The direct examination method depends upon the reliability of the species boundaries in the reference species. Cases of discordance between the boundaries of reference species and OTUs can arise in two ways. They can reflect errors between true species boundaries and those recognized by current taxonomy. In these cases, the reference species have been wrongly delineated, and a discordant OTU might reveal the true species boundary. Mistakes caused by taxonomic errors can be corrected when discovered. However, cases of discordance can also arise due to biological factors when a reference species corresponds with the true species boundary, but OTU delineation methods cannot recover it because of weaknesses in the analytical method or in the underlying data.

Taxonomic errors reflect the subjectivity that is often involved in drawing the line between two species. In addition to errors caused by insufficient knowledge of the focal taxa, taxonomic errors can arise through the use of unsuitable characters. For instance, an inappropriate reliance on wing venation led to oversplitting in one species complex of European elachistids [84]. As a general rule, the accuracy of species delineation improves as a particular group experiences recurrent study.

One major biological factor is the age of species. Young species tend to have unclear boundaries because processes such as hybridization and introgression are ongoing. As ecological differences can arise very rapidly via divergent selection [85,86], their use in species diagnosis can enhance the discovery of young species. Because DNA barcodes are single-locus data from the mitochondrial genome, they usually cannot recover OTUs which follow true species boundaries in cases where introgression is prevalent [87]. In general, the delineation of such species is challenging for all methods and a combination of several types of characters coupled with a careful sampling scheme is required (e.g., [88,89]). However, this approach is not an option for the first phase of species discovery when synoptic methods, such as DNA barcodes, are the best tools.

In addition to taxonomic and biological factors which influence the precision of the reference species, the sampling scheme can also impact the performance of the delineation methods. Optimally, a sufficiently large number of specimens covering the whole geographic distribution of each species would be included, and the study would include all known species of the focal clade. Unfortunately, this optimal scenario is rarely feasible, especially when a high number of poorly-known species is included. However, it should be noted that including a small number of specimens per species and/or sampling only distantly related species may artificially improve the congruence between species and OTUs. Restricted geographic sampling can have a similar effect, although the impact of geographical distance on the intraspecific variance has shown taxon-dependence [90–92]. Hausmann *et al*. [93] studied the performance of the BIN system in the well-studied geometrid moths with a large-scale sampling scheme, covering most parts of Europe. They reported rather poor performance (67% MATCHES), but concluded that many cases of discordance reflected flaws in the current taxonomy rather than problems with the method. Restricted intraspecific sampling can also raise the number of singletons in the data, which might affect the method performance. However, no effect of this type was discovered here (see Fig 8) or in a previous study evaluating the performance of GMYC [48].

Eight species of Gelechiinae and 32 species of Elachistinae were delineated differently in this study than in current taxonomy (i.e., three or four out of four methods produced discordant results, see species marked with asterisks in Table 1). To evaluate the effect of the sampling scheme, these species were studied for their number of BINs (i.e., OTUs delineated by the RESL algorithm) on BOLD (results in S4 Table). As these BINs are based on all sequence data on BOLD, the sampling effort was increased for most gelechiine species. No conflicts between the results in this study and the BINs were revealed. As additional specimens were available for seven of eight species from various parts of Europe and North America, sampling-based error is an unlikely explanation. Instead, it is possible that these reference species, especially the five species which were SPLIT in this study, each reflect a case of overlooked species and, thus, the discordance observed between current species boundaries and OTUs reflects a taxonomic error. However, two gelechiine species (*Scrobipalpa artemisiella* and *S*. *stangei*) were MERGED in the same OTU here as well as in the same BIN on BOLD. This case of discordance might be due to a biological factor as these species can easily be separated by morphology and have different life histories. Sampling-based error could not be evaluated for one gelechiine species (*Scrobipalpa bryophiloides*) as no additional sequences were available.

The examination of the BIN records on BOLD provided only a few additional records for the Australian Elachistinae so the effect of sampling could not be evaluated. However, many discordant results for the Elachistinae species were MERGES, leading to a higher number of specimens per OTU. As a consequence, the overall sample size per OTU was larger for the Elachistinae than the Gelechiinae. Seventeen elachistine species formed three groups with highly discordant results between the reference species and the delineated OTUs (see asterisks with numbers in Table 1). *E*. *lachnella* was included to this comparison, because it was MERGED in the same BIN with *E*. *nodosae*. This discordance between the result of this study and the BIN was due to one intermediate *E*. *nodosae* specimen on BOLD. As these three groups included 36, 12, and 10 sequences, sampling effort was not low so sampling-based error is unlikely. Instead, both biological and taxonomic factors may explain the observed discrepancy. These species were originally delineated based on ecological traits (in particular phenology, host plant selection and the shape of leaf mines that their larvae produce) which were correlated with small, but consistent morphological differences, a pattern compatible with their recent origin. Their young age is further supported by the distribution of pairwise genetic distances in Fig 2. However, as these species were described very recently (2011), they have not yet experienced critical re-examination so the species hypotheses cannot be considered as fully robust. As well, their young age might reflect taxonomical complications such as introgression which would make species boundaries difficult to interpret.

Fourteen *Elachista* species, which were originally delineated based on morphological differences, were MERGED with their sister species. In one case (*E*. *ophelma* and *E*. *catagma*), the representatives of each species formed a distinct subcluster within the shared OTU. Two other species (*E*. *gerasmia* and *E*. *physalodes*) showed a similar pattern, but one specimen was grouped with the subcluster otherwise comprised solely of its sister species. In three cases, the subcluster of one species was nested within its sister species (*E*. *anolba* and *E*. *averta*; *E*. *zophosema* and *E*. *litharga*; *E*. *stictifica* and *E*. *platysma*). Two pairs (*E*. *ophthalma* and *E*. nr. *ophthalma*; *E*. sp. ANICLK1 and *E*. sp. ANICLK3) included undescribed, but morphologically distinct species, which were MERGED with their sister species. These cases may also reflect both biological and taxonomic factors associated with recently diverged taxa. Only two *Elachista* species were designated as SPLITS (*E*. *carcharota* and *E*. *discina*). The split within *E*. *carcharota* was associated with large geographical distance (western vs. eastern Australia), the specimens were originally deemed conspecific because morphological differences were not apparent. *E*. *discina* was divided into two OTUs from sites in proximity. Both cases of SPLITS may reflect problems introduced by the small number of samples per species, but the possibility of overlooked cryptic species cannot be excluded.

### Method performance

Some general patterns in method performance were present regardless of the taxon. BIN, TCS with cut-off value 95%, and GMYC with Bayesian input trees and the single-threshold model produced similar results for both datasets (97–102 OTUs, 89–91% MATCHES for Gelechiinae; 81–96 OTUs, 62–67% MATCHES for Elachistinae). GMYC analyses based on Bayesian trees and BIN performed slightly better than TCS (95% cut-off), especially with the Elachistinae. As the performance of GMYC and BIN was similar for the Elachistinae, there was no evidence to support Zhang *et al*.’s [10] contention that tree-based approaches are superior for taxa with a narrow barcode gap.

The performance of GMYC was found to be very sensitive to the starting tree. UPGMA trees produced poor results with regard to both OTU count and composition (Figs 4–6, Table 3). Similar sensitivity has been reported in previous studies, which have tested this method with different trees [39,40,48,94]. Tang *et al*. [94] noted that starting trees transformed to ultrametric by *post hoc* branch smoothing (e.g., by employing function ‘chronopl’ in R) perform especially poorly. This feature complicates the use of GMYC with large datasets, because computationally expensive BEAST trees seem to be the only reliable option. Another noteworthy feature of GMYC is the weak performance of the multiple-threshold model which was also detected in a previous simulation study [21].

ABGD produced both the highest and some of the lowest percentages of MATCHES for both datasets. As different prior intraspecific divergence (*P*) values (when used with default parameters) lead ABGD to generate variable OTU counts, it would be optimal to choose a fixed *P*-value to gain just one result. *P* = 0.01 has been proposed [8], and this value performed well for the Gelechiinae (90–92% MATCHES), but poorly for the Elachistinae (50–59% MATCHES). We conclude that the adoption of one *P*-value can result in either high or low performance, depending on the focal group. Without a fixed *P*, ABGD generates a range of outcomes, meaning that the user must choose the ‘correct’ result, compromising the objectivity of this DNA-based method.

ABGD also showed considerable sensitivity to the distance metric adopted. The results with p-distance were most discordant, but K2P and JC also produced variable results for the Elachistinae. Similar discordance was observed in a study on Australian hypertrophine moths [17]. As the effect of distance metric on barcoding results is minor [95], the divergent OTU counts arising from the use of different distance metrics in ABGD seems to reflect a methodological problem. Puillandre *et al*. [8] have noted that ABGD requires 3–5 specimens per species for ideal performance, but this criterion is difficult to meet, especially if the number of taxa is unknown. As this issue has been discussed earlier [17], we only point out that our intraspecific sampling was mostly below this minimum level, but the general performance was still mainly congruent with the other tested methods.

### External morphology vs. DNA barcodes

Groupings based on external morphology has been the primary method for species delineation for centuries. The results from the morphological sorting in this study generated a low percentage of MATCHES for both subfamilies and a high proportion of MIXTURES, a particular challenge for subsequent taxonomic work. Although this test involved low sample sizes, it still provides an estimate of the relative efficacies of OTU designation via external morphology versus DNA-based methods. As with many other gelechioid moths, both elachistines and gelechiines are small and dull-colored, often lacking clearcut differences in external morphology. Furthermore, many elachistine species are sexually dimorphic [52], which might have contributed to the lower number of MATCHES for the Elachistinae. As the superfamily Gelechioidea includes many undescribed species, the need for efficient tools to expedite taxonomic workflows is of high importance. The present study reveals that the sole reliance on external morphology for the initial phase of taxonomic work will slow progress. We do expect that performance would have been improved if sorting had been done by an experienced gelechioid taxonomist, but this is not a general solution because many insect groups lack experts.

### Conclusions

Our results affirm the general effectiveness of current algorithmic methods for species delineation together with DNA barcodes as a tool for initial species discovery. Such analyses will be particularly useful for poorly-known groups with constrained external phenotypic variation. However, we urge careful selection of methods and parameters (and starting trees where applicable) as the same approach can produce results whose quality varies depending on the focal taxon, parameter values, and distance metrics. Furthermore, a parameter value which provides a high-quality outcome in one group can generate poor results for another. The combined use of several methods provides one way to obtain a more robust estimate of species boundaries [96]. Because the focal taxa are generally poorly known in studies aiming to delineate putative species, little information on evolutionary history is usually available. Examination of the width of the barcode gap with pairwise distances without *a priori* grouping does provide a preliminary estimate of the levels of divergence for the group under study, a potential aid to the interpretation of results.

Some authors have indicated that DNA barcode-based methods are not useful in the absence of prior knowledge on the focal group (e.g., [91]), but we disagree. Due to its speed, simplicity and objectivity, the analysis of DNA barcode data with species delineation methods is the most feasible tool for large-scale screening of poorly-known biodiversity. It provides an accelerated start for subsequent studies which can employ broader sampling and examine more characters.

## Supporting Information

S1 Table A table including BOLD Sample IDs, BOLD Process IDs, institutions and GenBank accessions. (XLSX)

S2 Table Comparison of the performance of four analytical methods (ABGD, BIN, GMYC, TCS) with singletons, ranked by the number of MATCHES. (DOCX)

S3 Table Comparison of the performance of four analytical methods (ABGD, BIN, GMYC, TCS) with monophyletic species, ranked by the number of MATCHES. (DOCX)

S4 Table Comparison of discordant OTUs (8 gelechiine and 33 elachistine species) and BINs on BOLD. *Elachista lachnella* is included, because it was merged in the same BIN with *E*. *nodosae*.
(XLSX)

S1 Appendix XML file for BEAST (Gelechiinae, Yule). (XML)

S2 Appendix XML file for BEAST (Gelechiinae, coalescent). (XML)

S3 Appendix XML file for BEAST (Elachistinae, Yule). (XML)

S4 Appendix XML file for BEAST (Elachistinae, coalescent). (XML)
