# Supplementary material for: Delineating Species with DNA Barcodes: A Case of Taxon Dependent Method Performance in Moths
Source: PLoS One. 2015 Apr 7;10(4):e0122481. doi: 10.1371/journal.pone.0122481 (PMC4406103; doi:10.1371/journal.pone.0122481)
Supplement: S2 Table — (DOCX) [file pone.0122481.s002.docx]

S6 Table. Comparison of the performance of four analytical methods (ABGD, BIN, GMYC, TCS) with singletons, ranked by the number of MATCHES. There are 36 outcomes for ABGD for the Gelechiinae (JC and K2P are combined as the results were identical) and 32 for the Elachistinae. Description of parameters and MATCH and MERGE categories are provided in the Material and Methods.

| **Dataset** | **Method** | **Parameters** |  | **MATCH** | **MERGE** |
| --- | --- | --- | --- | --- | --- |
| Gelechiinae | GMYC | UPGMA | multiple | 2 | 4 |
|  | GMYC | BEAST, coalescent | multiple | 3 | 3 |
|  | GMYC | BEAST, Yule | multiple | 4 | 2 |
|  | ABGD | p-distance, X=0.8 | P=0.0359* | 5 | 1 |
|  | ABGD | p-distance, X=1 | P=0.0359* | 5 | 1 |
|  | ABGD | p-distance, X=1 | P=0.0215 | 5 | 1 |
|  | ABGD | p-distance, X=1 | P=0.0129 | 5 | 1 |
|  | ABGD | p-distance, X=1 | P=0.00464 | 5 | 1 |
|  | ABGD | p-distance, X=1 | P=0.00278 | 5 | 1 |
|  | ABGD | p-distance, X=1 | P=0.001 | 5 | 1 |
|  | ABGD | JC, K2P, X=0.8 | P=0.0359* | 5 | 1 |
|  | ABGD | JC, K2P, X=1 | P=0.0359* | 5 | 1 |
|  | ABGD | JC, K2P, X=1 | P=0.0215 | 5 | 1 |
|  | ABGD | JC, K2P, X=1 | P=0.0129 | 5 | 1 |
|  | ABGD | JC, K2P, X=1 | P=0.00464 | 5 | 1 |
|  | ABGD | JC, K2P, X=1 | P=0.00278 | 5 | 1 |
|  | ABGD | JC, K2P, X=1 | P=0.001 | 5 | 1 |
|  | BIN |  |  | 6 | 0 |
|  | TCS |  | 90% | 6 | 0 |
|  | TCS |  | 91% | 6 | 0 |
|  | TCS |  | 92% | 6 | 0 |
|  | TCS |  | 93% | 6 | 0 |
|  | TCS |  | 94% | 6 | 0 |
|  | TCS |  | 95% | 6 | 0 |
|  | TCS |  | 96% | 6 | 0 |
|  | TCS |  | 97% | 6 | 0 |
|  | TCS |  | 98% | 6 | 0 |
|  | TCS |  | 99% | 6 | 0 |
|  | ABGD | p-distance, X=0.8 | P=0.0215 | 6 | 0 |
|  | ABGD | p-distance, X=0.8 | P=0.0129 | 6 | 0 |
|  | ABGD | p-distance, X=0.8 | P=0.00464 | 6 | 0 |
|  | ABGD | p-distance, X=0.8 | P=0.00278 | 6 | 0 |
|  | ABGD | p-distance, X=0.8 | P=0.001 | 6 | 0 |
|  | ABGD | JC, K2P, X=0.8 | P=0.0215 | 6 | 0 |
|  | ABGD | JC, K2P, X=0.8 | P=0.0129 | 6 | 0 |
|  | ABGD | JC, K2P, X=0.8 | P=0.00464 | 6 | 0 |
|  | ABGD | JC, K2P, X=0.8 | P=0.00278 | 6 | 0 |
|  | ABGD | JC, K2P, X=0.8 | P=0.001 | 6 | 0 |
|  | GMYC | UPGMA | single | 6 | 0 |
|  | GMYC | BEAST, coalescent | single | 6 | 0 |
|  | GMYC | BEAST, Yule | single | 6 | 0 |
| Elachistinae | ABGD | p-distance, X=0.8 | P=0.0215* | 9 | 25 |
|  | ABGD | p-distance, X=0.8 | P=0.0215 | 11 | 23 |
|  | ABGD | p-distance, X=0.8 | P=0.0129 | 14 | 20 |
|  | ABGD | p-distance, X=0.8 | P=0.00774 | 14 | 20 |
|  | ABGD | JC, X=0.8 | P=0.0215* | 20 | 14 |
|  | ABGD | JC, X=0.8 | P=0.0215 | 20 | 14 |
|  | ABGD | JC, X=0.8 | P=0.0129 | 20 | 14 |
|  | ABGD | JC, X=0.8 | P=0.00774 | 20 | 14 |
|  | ABGD | p-distance, X=0.8 | P=0.00464 | 22 | 12 |
|  | TCS |  | 90% | 23 | 11 |
|  | TCS |  | 91% | 23 | 11 |
|  | TCS |  | 92% | 23 | 11 |
|  | ABGD | K2P, X=0.8 | P=0.0129* | 23 | 11 |
|  | ABGD | K2P, X=0.8 | P=0.0129 | 23 | 11 |
|  | ABGD | K2P, X=0.8 | P=0.00464 | 23 | 11 |
|  | TCS |  | 93% | 24 | 10 |
|  | ABGD | JC, X=0.8 | P=0.00464 | 24 | 10 |
|  | TCS |  | 94% | 25 | 9 |
|  | TCS |  | 95% | 25 | 9 |
|  | BIN |  |  | 26 | 8 |
|  | TCS |  | 96% | 26 | 8 |
|  | TCS |  | 97% | 26 | 8 |
|  | GMYC | UPGMA | multiple | 26 | 8 |
|  | GMYC | BEAST, Yule | single | 28 | 6 |
|  | GMYC | BEAST, Yule | multiple | 28 | 6 |
|  | GMYC | BEAST, coalescent | multiple | 29 | 5 |
|  | TCS |  | 98% | 30 | 4 |
|  | TCS |  | 99% | 30 | 4 |
|  | ABGD | p-distance, X=0.8 | P=0.00278* | 30 | 4 |
|  | ABGD | p-distance, X=1.0 | P=0.00278* | 30 | 4 |
|  | ABGD | p-distance, X=1.0 | P=0.00278 | 30 | 4 |
|  | ABGD | JC, X=0.8 | P=0.00278* | 30 | 4 |
|  | ABGD | JC, X=1 | P=0.00278* | 30 | 4 |
|  | ABGD | JC, X=1 | P=0.00278 | 30 | 4 |
|  | ABGD | JC, X=1 | P=0.00167 | 30 | 4 |
|  | ABGD | K2P, X=0.8 | P=0.00278* | 30 | 4 |
|  | ABGD | K2P, X=1 | P=0.00278* | 30 | 4 |
|  | ABGD | K2P, X=1 | P=0.00278 | 30 | 4 |
|  | GMYC | BEAST, coalescent | single | 30 | 4 |
|  | ABGD | p-distance, X=0.8 | P=0.00278 | 31 | 3 |
|  | ABGD | p-distance, X=1.0 | P=0.001 | 31 | 3 |
|  | ABGD | JC, X=0.8 | P=0.00278 | 31 | 3 |
|  | ABGD | JC, X=1 | P=0.001 | 31 | 3 |
|  | ABGD | K2P, X=0.8 | P=0.00278 | 31 | 3 |
|  | ABGD | K2P, X=1 | P=0.001 | 31 | 3 |
|  | ABGD | p-distance, X=0.8 | P=0.001 | 32 | 2 |
|  | ABGD | JC, X=0.8 | P=0.001 | 32 | 2 |
|  | ABGD | K2P, X=0.8 | P=0.001 | 32 | 2 |
|  | GMYC | UPGMA | single | 32 | 2 |

BEAST: Bayesian gene tree reconstructed in BEAST, Yule: Yule tree prior, Coalescent: coalescent tree prior, Single: single threshold model, Multiple: multiple threshold model, JC: Jukes-Cantor substitution model, K2P: Kimura two parameter substitution model, X: relative gap width, P: prior intraspecific divergence value, *: initial partition
